# Molecular Mechanisms of Chondrocyte Hypertrophy Mediated by Physical Cues and Therapeutic Strategies in Osteoarthritis

**DOI:** 10.3390/ijms27020624

**Published:** 2026-01-08

**Authors:** Guang-Zhen Jin

**Affiliations:** Institute of Tissue Regeneration Engineering, Dankook University, Cheonan 31116, Republic of Korea; gzhjin2002@dankook.ac.kr; Tel.: +82-41-550-3082

**Keywords:** chondrocyte hypertrophy, biophysical cues, mechanotransduction, anti-hypertrophic therapeutic strategies, osteoarthritis

## Abstract

Osteoarthritis (OA) is a multifactorial degenerative joint disease in which aberrant mechanical cues act in concert with metabolic dysregulation and chronic low-grade inflammation, with chondrocyte hypertrophy representing a key pathological event driving cartilage degeneration. Alterations in extracellular matrix (ECM) properties—including mechanical loading, stiffness and viscoelasticity, topological organization, and surface chemistry—regulate hypertrophic differentiation and matrix degradation in a zone-, stage-, and scale-dependent manner. Microscale measurements often reveal localized stiffening in superficial zones during early OA, whereas bulk tissue testing can show softening or heterogeneous changes in deeper zones or advanced stages, highlighting the context-dependent nature of ECM mechanics. These biophysical signals are sensed by integrin-based adhesion complexes, primary cilia, mechanosensitive ion channels (TRP/Piezo), and the actin cytoskeleton–nucleus continuum, and are transduced into intracellular pathways with zone- and stage-specific effects, governing chondrocyte fate under physiological and osteoarthritic conditions. Mechanism-based anti-hypertrophic strategies include biomimetic scaffold design for focal defects, dynamic mechanical stimulation targeting early OA, and multimodal approaches integrating mechanical cues with biochemical factors, gene modulation, drug delivery, or cell-based therapies. Collectively, this review provides an integrated mechanobiological framework for understanding cartilage degeneration and highlights emerging opportunities for disease-modifying interventions targeting chondrocyte hypertrophy.

## 1. Introduction

Chondrocyte hypertrophy plays a pivotal role in both the physiological development and pathological degeneration of articular cartilage. During skeletal development, hypertrophic differentiation of chondrocytes is an essential step in endochondral ossification, characterized by increased cell volume and the coordinated upregulation of hypertrophic markers such as type X collagen, RUNX2, and matrix metalloproteinases (MMPs), alongside the gradual suppression of SOX9 and type II collagen expression, ultimately enabling cartilage mineralization and replacement by bone tissue [1,2,3,4]. In adult articular cartilage, aberrant reactivation of hypertrophic programs is widely recognized as a hallmark of osteoarthritis (OA) pathogenesis and is likely a major contributor to progressive cartilage degeneration [1,2,3].

Recent transcriptomic studies have revealed substantial heterogeneity among hypertrophic chondrocytes in OA cartilage. Using single-cell RNA sequencing, Huang et al. identified two terminally differentiated hypertrophic chondrocyte subpopulations, termed HTC-1 and HTC-2, which exhibit distinct transcriptional programs and divergent cell fate trajectories [5]. HTC-1 represents an apoptosis- and stress-associated hypertrophic population that is markedly expanded in OA cartilage and is enriched for genes involved in extracellular matrix (ECM) degradation, vascular development, and pathological ossification, whereas HTC-2 is more closely linked to skeletal development and osteogenic differentiation pathways. Here, “abnormally hypertrophic chondrocytes” refers to chondrocytes in OA cartilage that exhibit excessive hypertrophic differentiation compared with healthy cartilage, characterized by overexpression of matrix-degrading enzymes (e.g., MMP-13), enhanced alkaline phosphatase (ALP) activity, and increased propensity for pathological calcification. Consistent with transcriptomic features, these cells have been reported to secrete excessive matrix-degrading enzymes and to promote pathological calcification, contributing to cartilage matrix breakdown and OA progression [6]. From a mechanobiological perspective, these distinct hypertrophic subpopulations likely reflect differential cellular responses to altered mechanical loading and matrix microenvironmental cues in osteoarthritic cartilage.

Accumulating evidence indicates that alterations in the physical properties of the cartilage ECM are critical drivers of chondrocyte phenotypic instability in OA. Biophysical parameters such as matrix stiffness, viscoelasticity, porosity, and nano- to microscale topography are dynamically remodeled during OA progression and can profoundly influence chondrocyte behavior [7,8]. Observations of ECM mechanical changes in OA are highly context-dependent, varying with cartilage zone, disease stage, and measurement scale. For example, atomic force microscopy (AFM) measurements have revealed that superficial cartilage may exhibit softening, whereas deeper zones or bulk tissue can show stiffening, reflecting both local microstructural alterations and overall matrix remodeling [9,10]. Some studies report early softening associated with proteoglycan loss, while late-stage OA often involves stiffening due to collagen crosslinking, calcification, or subchondral bone changes [10,11,12]. These heterogeneous and scale-dependent changes in ECM mechanics likely provide distinct mechanotransduction cues to chondrocytes, influencing hypertrophic differentiation and sustaining a pro-degenerative cellular state [9,10,11,12,13].

These physical cues are persistent, spatially organized, and context-dependent, and are primarily sensed by chondrocytes through mechanotransduction mechanisms involving integrins, mechanosensitive ion channels, and cytoskeletal–nuclear coupling [14]. OA-associated ECM remodeling, including heterogeneous stiffness changes, microstructural disruption, and altered load distribution, generates aberrant mechanical signals that can favor hypertrophic differentiation and maintain a pro-degenerative cellular state [9,10,11,12,13]. Accordingly, targeting ECM-derived physical cues represents a promising strategy for modulating chondrocyte hypertrophy and potentially intervening in OA progression at an early, disease-modifying level [15].

Although substantial progress has been made in elucidating intracellular mechanotransduction pathways that regulate chondrocyte hypertrophy, including Ca^2+^ signaling, the integrin–FAK–RhoA/ROCK axis, and YAP/TAZ-mediated transcriptional control, important knowledge gaps remain regarding how these pathways are integrated within the inflammatory microenvironment of osteoarthritic cartilage. Pro-inflammatory mediators, such as cytokines and chemokines, activate signaling cascades including NF-κB, which regulate matrix catabolism, cell survival, and differentiation in chondrocytes [16]. However, how inflammatory signaling reshapes mechanosensitive responses and cooperates with physical cues to drive hypertrophic fate commitment remains incompletely understood. Elucidating this mechanochemical crosstalk is essential for the development of precise, mechanism-based therapeutic interventions for OA.

In this review, we systematically summarize the molecular mechanisms by which ECM-derived physical cues regulate chondrocyte hypertrophy and matrix degradation through mechanotransduction pathways, with particular emphasis on their relevance to OA pathogenesis. We further discuss three major ECM-oriented intervention strategies, including biomimetic scaffold-based microenvironmental optimization, restoration of physiological mechanosensing via dynamic mechanical stimulation, and integrated multimodal approaches that combine physical cues with biochemical factors, gene modulation, drug delivery, or cell-based therapies. By bridging mechanobiological insights with molecular mechanisms of OA, this review aims to provide a theoretical framework and mechanistic guidance that may inform the rational design and future exploration of innovative anti-hypertrophic intervention strategies in OA.

## 2. Pericellular Matrix and Cell Membrane Mechanosensors in Chondrocyte Mechanotransduction

**Figure 1:**
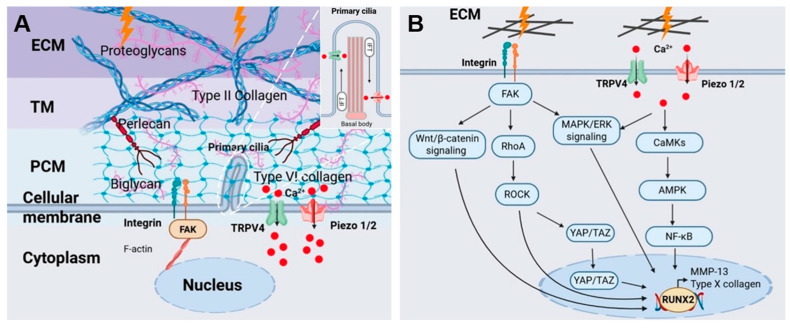
Mechanotransduction at the chondrocyte PCM under mechanical stress: (**A**) Schematic representation of the PCM and membrane-associated mechanosensors. The ECM comprises the PCM, TM, and interterritorial matrix. The PCM is enriched in perlecan, biglycan, and type VI collagen and, together with key mechanosensors including mechanosensitive Ca^2+^ channels (TRPV4, Piezo1/2), integrins, and primary cilia, forms the core mechanotransduction interface. The inset highlights the microtubule-based cytoskeleton of the primary cilium and the bidirectional trafficking of signaling molecules mediated by IFT. (**B**) Schematic illustrating how mechanosensitive Ca^2+^ channels and integrin-mediated signaling pathways coordinately regulate chondrocyte hypertrophy and ECM degradation under pathological mechanical stress. Abbreviations: PCM, pericellular matrix; TM, territorial matrix; ECM, extracellular matrix; IFT, intraflagellar transport; TRPV4, transient receptor potential vanilloid 4; F-actin, filamentous actin; FAK, focal adhesion kinase; Piezo1/2, piezo-type mechanosensitive ion channel component 1/2; YAP/TAZ, Yes-associated protein/transcriptional coactivator with PDZ-binding motif; NF-κB, nuclear factor-κB; CaMKs, Ca^2+^/calmodulin-dependent protein kinases; AMPK, AMP-activated protein kinase; MMP-13, matrix metalloproteinase-13; RhoA/ROCK, RhoA/Rho-associated coiled-coil kinase; RUNX2, Runt-related transcription factor 2. Figure created with BioRender (licensed).

Chondrocytes reside within a highly specialized mechanical microenvironment in articular cartilage. The pericellular matrix (PCM), together with mechanosensitive structures on the cell membrane, including integrins, ion channels, and primary cilia, forms a functional mechanosensory unit that enables chondrocytes to perceive, integrate, and respond to physical cues from the ECM (Figure 1). This integrated system plays a central role in maintaining cartilage homeostasis and in driving pathological phenotypic changes such as hypertrophy during OA progression.

### 2.1. The PCM as a Mechanical Buffer and Signal Modulator

The PCM is a specialized microenvironment that surrounds individual chondrocytes with a thickness of approximately 1 to 5 μm. It is enriched in proteoglycans such as perlecan, aggrecan, hyaluronic acid, and biglycan, as well as type IX and type VI collagens, with type VI collagen serving as a defining structural component of the PCM [17,18]. As summarized in prior studies, interactions between perlecan and type VI collagen are critical for stabilizing PCM architecture and enabling bidirectional mechanical communication between chondrocytes and the surrounding ECM [17].

Compared with the territorial and interterritorial ECM, the PCM exhibits markedly lower stiffness. AFM studies have demonstrated that the elastic modulus of the inner PCM layer is approximately 27.5 ± 8.8 kPa, whereas the surrounding ECM reaches values of approximately 151.4 ± 38.2 kPa [19]. It should be noted that AFM primarily reflects local micromechanical properties rather than bulk ECM stiffness; thus, PCM mechanics are highly context-dependent, varying with cartilage zone, OA stage, and measurement scale.

This compliant mechanical property allows the PCM to function as a mechanical buffer that attenuates excessive extracellular forces and modulates the magnitude and spatial distribution of mechanical signals in a zone-, stage-, and scale-dependent manner. For instance, its protective effect may differ between superficial versus deep zones and early versus late OA cartilage, as suggested in reviews [17,18]. By regulating local stiffness and strain transfer, the PCM plays a critical role in fine-tuning mechanotransduction and preserving chondrocyte phenotype. Disruption of PCM composition or mechanics may amplify pathological mechanical signaling and contribute to hypertrophy and cartilage degeneration [17,19].

### 2.2. Integrin-Mediated Mechanotransduction and YAP/TAZ Signaling in Chondrocyte Fate Regulation

Integrins are transmembrane adhesion receptors that serve as key mechanosensors by linking ECM components to the intracellular cytoskeleton [20]. Upon binding to ligands such as collagen or fibronectin, integrins cluster to form focal adhesions and activate downstream signaling pathways, most notably the FAK–RhoA/ROCK cascade. This activation enhances actomyosin contractility and cytoskeletal tension, allowing mechanical forces to be transmitted from the cell surface to the nucleus [20].

Mechanical signals conveyed through integrins promote nuclear translocation of the transcriptional co-activators YAP and TAZ, which play central roles in regulating chondrocyte differentiation and hypertrophic progression [21]. As summarized in prior reviews, YAP/TAZ modulate the activity of lineage-specific transcription factors, including SOX9 and RUNX2, thereby exerting context-dependent control over chondrogenesis and hypertrophy. Under physiological or homeostatic conditions, reduced YAP and TAZ activity is frequently associated with increased expression of hypertrophic markers such as RUNX2, type X collagen, and MMP-13, suggesting that the Hippo–YAP/TAZ pathway functions as a critical brake on premature chondrocyte hypertrophy [22,23,24].

In addition to regulating transcriptional programs, integrin-mediated mechanotransduction influences cell cycle progression, apoptosis, and cellular metabolism [25,26]. While increased ECM stiffness generally promotes YAP/TAZ nuclear accumulation, the magnitude and direction of the effect depend on cartilage zone, PCM properties, OA stage, and measurement scale (AFM vs. bulk tissue) [21,27]. Reduced stiffness or inhibition of integrin signaling decreases cytoskeletal tension and limits YAP/TAZ activity [28,29]. These observations support a context-sensitive model in which basal YAP/TAZ signaling maintains homeostasis and suppresses hypertrophic differentiation [22,23,24].

Integrin signaling further interacts with other mechanoresponsive pathways, including MAPK–ERK, Wnt/β-catenin, and TGF-β signaling, forming a complex mechano-chemical network [30,31]. This multi-layered integration allows chondrocytes to translate mechanical cues into transcriptional responses that are zone-, stage-, and scale-dependent [21,30].

### 2.3. Mechanosensitive Calcium Channels in Chondrocyte Inflammation and Matrix Remodeling

Mechanosensitive calcium channels constitute a central component of chondrocyte mechanotransduction, including Piezo1, Piezo2, and TRPV4, which respond to ECM stiffness, osmotic pressure, and mechanical loading by mediating calcium influx and activating downstream signaling pathways [32,33,34,35,36].

TRPV4 exhibits context-dependent functions, promoting anabolic signaling and maintaining ECM homeostasis under physiological loading [32]. However, under pathological conditions such as mechanical overload, altered viscoelasticity, osmotic stress, or inflammatory exposure, TRPV4 signaling modulates chondrocyte responses in a zone-, stage-, and scale-dependent manner via pathways such as CaMKK, AMPK, and NF-κB [37,38,39].

Piezo1 is highly sensitive to ECM stiffness and mechanical stress. Pro-inflammatory cytokines, such as IL-1β, enhance Piezo1 mechanosensitivity, forming a feed-forward loop that amplifies pathological mechanical signaling and accelerates OA progression [33]. Recent studies demonstrate crosstalk between Piezo1 and TRPV4 that modulates gene expression in human chondrocytes [40]. It is important to note that responses can differ between in vitro models and ex vivo explants, highlighting measurement-scale dependence.

Calcium influx mediated by these channels influences inflammatory and catabolic pathways, including NF-κB and MAPK, regulating cytokine and MMP-13 expression [41,42,43,44]. Activation of Piezo1 and TRPV4 is context-dependent, with zone-, stage-, and scale-specific effects. Piezo1/2 cooperation confers high-strain mechanosensitivity, and dysregulation contributes to OA pathogenesis [36,41]. Excessive mechanical stress or ECM stiffening upregulates Piezo1, especially in late-stage OA deep zones, and is associated with senescence, mitochondrial autophagy dysfunction, and impaired matrix synthesis [34,40,45].

### 2.4. Primary Cilia as Integrative Mechanosensors in Chondrocyte Homeostasis and OA

Primary cilia are microtubule-based organelles projecting from most chondrocytes, functioning as mechanosensors and signaling hubs [46,47]. They integrate mechanical, osmotic, and biochemical signals, regulating Hedgehog, Wnt/β-catenin, and calcium pathways to control proliferation, differentiation, and matrix metabolism.

Changes in extracellular osmotic pressure affect ciliary structure. Hyperosmotic conditions shorten primary cilia, linking ECM osmotic homeostasis to mechanosensing [46]. Mechanical loading dynamically alters cilia prevalence and length, demonstrating active response rather than passive sensing [47].

Ciliary length correlates with signaling competence; overly short or disorganized cilia impair mechanosensing, whereas elongated cilia may distort signal transduction in a zone-, stage-, and species-dependent manner [46,47]. OA cartilage exhibits heterogeneous ciliary abnormalities due to regional loading and inflammatory milieu. Review evidence highlights the need to interpret ciliary function and stiffness effects in a precise experimental and physiological context [46,47].

Disruption of ciliary structure compromises mechanosensory integration, perturbs mechanical–inflammatory balance, and accelerates cartilage degeneration. Future studies quantifying ciliary structure–function relationships will elucidate how physical cues regulate pathology and may reveal novel OA targets.

## 3. ECM Biophysical Cues as Regulators of Chondrocyte Hypertrophy in OA

### 3.1. Mechanical Cues in the Osteoarthritic Cartilage Microenvironment

Mechanical cues within the osteoarthritic cartilage microenvironment exert multifaceted effects on chondrocytes. Changes in extracellular mechanical forces can alter chondrocyte shape, generate uneven stress distributions, and compromise matrix integrity, collectively influencing cell behavior and tissue homeostasis. Moreover, joint movement produces interstitial fluid flow around chondrocytes, generating shear stress signals that modulate cellular activity, including metabolism, proliferation, and differentiation. These mechanical and fluid-derived cues are sensed by mechanosensitive structures such as integrins, primary cilia, and ion channels, which transduce signals into intracellular biochemical responses in a zone-, stage-, and scale-dependent manner. Collectively, these mechanisms form the foundational context for subsequent discussions of ECM stiffness, viscoelasticity, porosity, topography, and surface cues, providing a coherent framework linking biophysical forces to chondrocyte hypertrophy and matrix remodeling in OA.

#### 3.1.1. Stiffness

The stiffness of the articular cartilage ECM exhibits pronounced scale-, region-, and OA stage-dependent variations, both physiologically and pathologically. At the tissue scale, the compressive modulus of healthy cartilage typically ranges from several hundred kilopascals to the megapascal range, as determined by macroscopic compression testing. Such bulk measurements primarily reflect load-bearing capacity and may not represent local micromechanical environments sensed by individual chondrocytes [48,49].

In contrast, stiffness of the PCM and local ECM regions, measured using AFM or micromechanical testing, is substantially lower, generally spanning several kilopascals to a few hundred kilopascals depending on measurement technique, cartilage zone, tissue source, and disease stage [50,51,52]. These locally compliant regions critically influence chondrocyte mechanosensing and downstream signaling, emphasizing the importance of specifying measurement scale and cartilage zone when interpreting stiffness data.

Under osteoarthritic conditions, the ECM undergoes context-dependent stiffening or softening, varying by cartilage zone and OA stage. Early OA may show localized softening in superficial zones due to proteoglycan loss, whereas late-stage OA often involves stiffening in deeper zones from collagen crosslinking and calcification [53,54]. Chondrocytes perceive ECM stiffness changes primarily via integrin-mediated adhesion complexes, activating downstream RhoA/ROCK signaling and influencing YAP/TAZ nuclear translocation in a zone- and stage-dependent manner [15,21]. This clarifies that statements about “stiffening in OA” must be qualified according to measurement context, cartilage zone, and disease stage.

#### 3.1.2. Viscoelasticity

Healthy articular cartilage displays pronounced viscoelastic behavior arising from the tensile properties of the collagen fiber network and the osmotic swelling pressure generated by proteoglycan–water interactions [48,49]. Proteoglycans, particularly aggrecan, regulate osmotic balance and time-dependent mechanical responses. Degradation of aggrecan by ADAMTS5 releases chondroitin sulfate chains, which can influence chondrocyte function [55]. Chondroitin sulfate incorporation into hyaluronic acid-based hydrogels can suppress pro-inflammatory cytokines and catabolic enzymes under inflammatory conditions [56].

Viscoelasticity enables cartilage to dissipate mechanical energy and buffer repetitive compressive and shear loads, preserving tissue integrity. In OA, proteoglycan loss compromises hydration and osmotic regulation, while collagen crosslinking and fiber disorganization reduce matrix compliance. These changes impair stress relaxation and hysteresis, transmitting mechanical loads more directly to chondrocytes in a zone-, stage-, and scale-dependent manner. Enhanced mechanical transmission can strengthen integrin–cytoskeleton coupling and activate mechanosensitive calcium channels (e.g., Piezo1, TRPV4), potentially involving NF-κB–associated pathways implicated in hypertrophic differentiation and matrix catabolism, including type X collagen and MMP-13 upregulation [32,57,58,59]. Biomimetic hydrogels recapitulating healthy cartilage viscoelasticity can partially restore chondrocyte homeostasis, suppress MMP-13 expression, and delay hypertrophic progression [58,59]. These observations are consistent with mechanistic evidence linking primary cilia-mediated mechanosensing to apoptosis and matrix metabolism [60,61].

### 3.2. Structural Cues and Microenvironmental Remodeling

#### 3.2.1. Porosity and Pore Size

Native articular cartilage exhibits a highly organized 3D porous architecture with regionally graded porosity and pore size distribution. PCM and surrounding ECM contain nano- to submicron-scale pores, supporting slow diffusion of oxygen and nutrients while establishing localized hypoxic gradients essential for maintaining chondrocyte metabolic homeostasis [48,50,62].

During OA progression, cartilage permeability and solute transport pathways undergo substantial remodeling. Alterations in hydraulic permeability and diffusivity disrupt oxygen and nutrient transport, destabilizing the microenvironment [63,64]. Advanced ECM degeneration often leads to pronounced hypoxia, impairing chondrocyte function in a zone- and stage-dependent manner [65]. Primary cilia integrate mechanical and osmotic cues, linking ECM changes to intracellular signaling that influences hypertrophic differentiation and matrix turnover [60,61].

HIF-1α is typically stabilized under physiological hypoxia, exerting protective effects by suppressing NF-κB activation or promoting autophagy, thereby reducing MMP-13 expression [66,67]. In contrast, HIF-2α acts as a key catabolic mediator in OA cartilage, inducing C/EBPβ and synergizing with RUNX2 to enhance MMP-13 transcription and degeneration [68,69]. The balance between HIF-1α and HIF-2α responses is highly context-dependent, reflecting local oxygen tension, mechanical environment, and cartilage zone.

In cartilage tissue engineering, scaffold pore sizes are typically designed within 100–300 μm to optimize chondrocyte adhesion, migration, and ECM synthesis [48,49]. However, optimal pore size is context-dependent, varying by cell type, scaffold material, mechanical loading, cartilage zone, and stage of tissue maturation. Excessively small pores may restrict cell infiltration and nutrient diffusion, whereas overly large pores may reduce adhesion area and mechanical support. Numerous studies demonstrate that scaffolds with high porosity and interconnectivity improve nutrient transport and ECM deposition [70,71]. Collagen scaffolds with 150–250 μm pores have been shown to enhance type II collagen and aggrecan expression, but optimal dimensions may differ between superficial vs. deep zones and early vs. late-stage constructs [72,73,74]. Functional cues, such as MMP-responsive hydrogels, may further preserve ECM and delay OA-like phenotypic progression in a stage- and zone-specific manner [58,59].

#### 3.2.2. Topography

The native cartilage ECM displays highly organized nano- to microscale topographical features, including horizontally aligned fibers in the superficial zone, randomized networks in the middle zone, and vertically oriented fibers in the deep zone. These zonal architectures regulate cell polarity, focal adhesion distribution, and cytoskeletal organization, thereby influencing chondrocyte phenotype [74,75]. In OA, ECM topography becomes increasingly disorganized, with disrupted fiber alignment and increased surface irregularity, impairing mechanosensing and spatial guidance in a zone- and stage-dependent manner [74,76].

Altered topographical cues modulate Rho family GTPases (RhoA, Rac1, Cdc42). Hyperactivation of RhoA/ROCK enhances stress fiber formation and focal adhesion maturation, promoting hypertrophy and catabolic gene expression (type X collagen, MMP-13) [77,78]. Dysregulation of Rac1 and Cdc42 further disrupts cytoskeletal dynamics, reinforcing OA-associated phenotypes. These effects are context-dependent, varying with cartilage zone, local stiffness, and OA stage.

### 3.3. Surface Cues and Cell Matrix Interactions

#### 3.3.1. Roughness

Surface roughness influences cell–matrix interactions. Moderate roughness supports stable integrin-mediated focal adhesion formation and mechanotransduction. Excessive micro- or nanoscale roughness alters contact geometry and local tension, promoting integrin clustering, focal adhesion maturation, and activation of mechanotransduction pathways such as FAK and ERK. Materials science studies demonstrate that hierarchical micro- and nanoscale roughness can activate FAK-MAPK and ILK-β-catenin signaling and influence MSC differentiation [79]. In OA, collagen degradation and fissure formation increase surface roughness, and integrin αvβ3 expression is upregulated, amplifying inflammatory mediator production and matrix degradation via FAK-dependent ERK signaling [80,81]. While surface roughness alone may not fully trigger MMP-13 upregulation, altered roughness plausibly promotes integrin activation and downstream hypertrophic/catabolic signaling in a context-dependent manner.

#### 3.3.2. Charge Distribution

Glycosaminoglycans (GAGs) are a major component of the cartilage ECM and carry fixed negative charges that interact with cations such as sodium ions, regulating osmotic swelling and tissue mechanical properties [82,83,84]. Evidence indicates that in OA, GAG degradation reduces fixed charge density, thereby altering the electrochemical and mechanical microenvironment of the matrix [33,40,85,86]. These changes may indirectly influence mechanosensitive calcium channels in chondrocytes, including TRPV4 and Piezo family channels, modulating intracellular calcium dynamics [33,40,85,86]. Perturbed calcium signaling can activate calcium-dependent proteases such as calpains, which have been implicated in ECM protein cleavage and cartilage degradation [87].

Overall, current evidence supports a biologically plausible pathway in which GAG loss alters electrochemical and mechanical cues, indirectly shaping chondrocyte calcium signaling and contributing to matrix degeneration. Several steps within this cascade still require direct in situ or in vivo experimental validation.

### 3.4. Integrative Summary of Mechanotransduction in OA Chondrocytes

To provide an at-a-glance overview of ECM biophysical cues, their mechanosensors, downstream signaling, and hypertrophic/catabolic outputs, we include the following integrative table (Table 1):

## 4. Mechanotransduction Pathways Driving Hypertrophy Under Physical Cues

**Figure 2:**
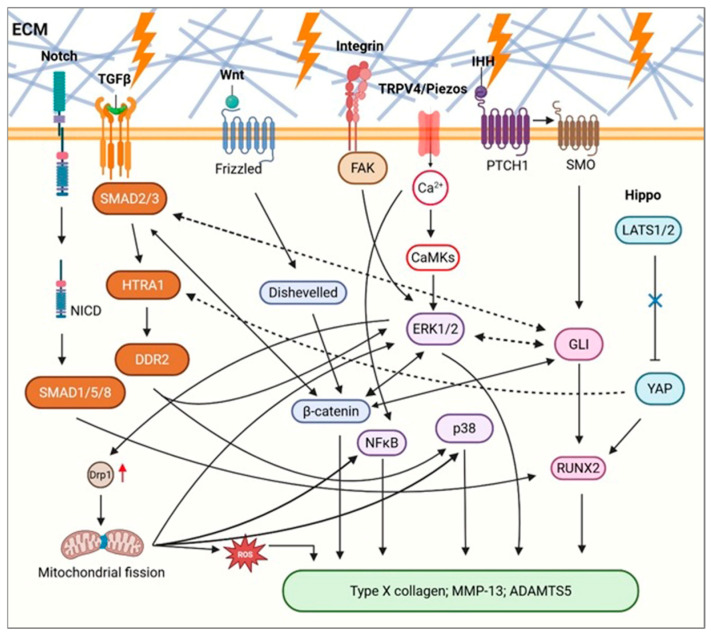
Crosstalk among key signaling pathways regulating chondrocyte hypertrophy under pathological mechanical stress. Schematic illustration of the mechanisms through which the crosstalk among the IHH, Wnt/β-catenin, MAPK/ERK, TGF-β, Hippo/YAP, and Notch signaling pathways coordinately regulates chondrocyte hypertrophy and ECM degradation under pathological mechanical stress. Solid arrows indicate signaling pathways supported by direct experimental evidence, whereas dashed arrows denote indirect or context-dependent interactions. Abbreviations: ECM, extracellular matrix; NICD, Notch intracellular domain; SMAD, SMAD family member; HTRA1, high-temperature requirement A serine peptidase 1; DDR2, discoidin domain receptor tyrosine kinase 2; Drp1, dynamin-related protein 1; ROS, reactive oxygen species; TGF-β, transforming growth factor-β; Wnt, Wnt signaling pathway; FAK, focal adhesion kinase; NF-κB, nuclear factor-κB; Ca^2+^, calcium ion; CaMKs, Ca^2+^/calmodulin-dependent protein kinases; ERK1/2, extracellular signal-regulated kinase 1/2; p38, p38 mitogen-activated protein kinase; TRPV4, transient receptor potential vanilloid 4; Piezos, Piezo-type mechanosensitive ion channels; IHH, Indian hedgehog; PTCH1, Patched 1; SMO, Smoothened; GLI, GLI family zinc finger; RUNX2, Runt-related transcription factor 2; Hippo, Hippo signaling pathway; LATS1/2, Large tumor suppressor kinase 1/2; YAP, Yes-associated protein; MMP-13, matrix metalloproteinase-13; ADAMTS5, A disintegrin and metalloproteinase with thrombospondin motifs 5. Figure created with BioRender (licensed).

Mechanosensors activate multiple signaling pathways, including Indian Hedgehog (IHH), Wnt/β-catenin, MAPK–ERK, TGF-β/SMAD, Hippo/YAP, and Notch, forming a complex regulatory network that integrates mechanical stimuli to control chondrocyte proliferation, differentiation, matrix metabolism, and tissue homeostasis. Crosstalk among these pathways allows precise decoding of mechanical signals, and their dysregulation promotes chondrocyte hypertrophy and matrix degradation, contributing critically to OA onset and progression (Figure 2).

### 4.1. IHH Pathway

The IHH signaling pathway regulates endochondral ossification and growth plate development by controlling chondrocyte proliferation, hypertrophy, and differentiation [5,88]. IHH binds to PTCH1, relieving SMO inhibition and activating GLI transcription factors, which cooperate with RUNX2 and SMADs to drive downstream gene expression, including type X collagen [88,89]. RUNX2 further mediates hypertrophic chondrocyte-driven MMP-13 expression, contributing to matrix remodeling [90]. Conditional deletion of IHH in cartilage reduces MMP-13 and type X collagen in OA models [91].

IHH signaling is highly mechanosensitive, with primary cilia essential for proper signal transduction. Cyclic tensile strain upregulates IHH, activates PTCH1/GLI, and increases ADAMTS5 in a cilium-dependent manner [92]. Aurora A kinase disrupts ciliary integrity through axonemal destabilization, explaining stress-induced cilia loss [93]. Chondrocyte dedifferentiation is associated with cilia loss and attenuated Hedgehog signaling, a phenomenon recapitulated by cyclic strain [94]. In OA, primary cilia abnormalities, including shortening, reduced number, or disassembly, correlate with sustained IHH activation, although regional heterogeneity and disease stage influence these patterns [95,96].

Restoring ciliary structure represents a potential therapeutic strategy. Lithium chloride extends cilia length and suppresses Hedgehog signaling (PTCH1, GLI1), mitigating overactive IHH–GLI signaling [97]. IFT protein deficiencies (e.g., Kif3a, IFT88) disrupt cilia and exacerbate early OA-like pathology [96,98]. These findings suggest that combined ciliary restoration and Hedgehog pathway modulation may be effective in OA intervention.

### 4.2. Wnt/β-Catenin Pathway

The Wnt/β-catenin signaling pathway is a central regulator of chondrocyte proliferation, differentiation, and phenotypic maintenance, with spatiotemporal control critical during cartilage homeostasis and disease. Mechanosensitive structures, particularly primary cilia, modulate Wnt/β-catenin signaling in a context-dependent manner, linking mechanical cues to hypertrophic and catabolic responses [99,100]. As summarized in a previous review [100], ciliary integrity is essential for proper Wnt signal transduction under mechanical loading.

Haycraft et al. demonstrated that intraflagellar transport protein 88 (IFT88) is essential for primary cilium assembly and function. Conditional deletion of Ift88 in Col2α1-Cre; Ift88^fl/n mice resulted in loss of primary cilia, disorganized chondrocyte maturation, and impaired endochondral ossification [101]. Although Hedgehog signaling is a primary downstream effector, ciliary loss may indirectly perturb multiple pathways, including Wnt/β-catenin. Consistently, McGlashan et al. (2008) reported that the proportion of chondrocytes bearing primary cilia increased with OA progression in bovine cartilage, accompanied by elongation of ciliary length [95].

Canonical Wnt/β-catenin signaling promotes chondrocyte hypertrophy and ECM remodeling. β-Catenin activation induces hypertrophic markers such as type X collagen and upregulates catabolic enzymes, including MMP-13 and ADAMTS5 [102,103,104]. In vitro and in vivo studies indicate that mechanical loading, ECM stiffness, and cilium integrity modulate these responses, emphasizing context-dependence across cartilage zones and disease stages. Pharmacological stabilization of β-catenin enhances MMP-13 and ADAMTS5 expression, whereas inhibition attenuates these catabolic effects [103]. Conditional activation of β-catenin in adult articular chondrocytes induces an OA-like phenotype characterized by elevated MMP-13, ADAMTS5, and type X collagen [104].

Crosstalk between IHH and Wnt/β-catenin signaling refines mechanically regulated chondrocyte differentiation. Primary cilium-associated signaling coordinates IHH–Wnt interactions, with Wnt/β-catenin promoting hypertrophy and upregulating IHH, reinforcing hypertrophic programs. Conversely, IHH reduces Gli3 repressor levels, relieving suppression of β-catenin–TCF/LEF activity [105,106]. IHH can also drive periarticular chondrocytes toward hypertrophy independently of PTHrP [107]. These interactions illustrate a mechanosensitive Wnt–IHH network integrating ECM stiffness and loading cues to regulate chondrocyte hypertrophy and ECM degradation in OA.

### 4.3. MAPK–ERK Pathway

The MAPK–ERK pathway integrates external physical and chemical cues, including mechanical stress [108]. Mechanical forces activate ERK1/2 and regulate matrix-related genes such as type II collagen and MMP-13, influencing differentiation, metabolism, stress responses, and inflammation [109]. Mechanosensitive TRPV4 channels mediate Ca^2+^ influx, potentially linking mechanical stimuli to ERK activation via a cilium–TRPV4–Ca^2+^–ERK axis [110,111,112,113,114]. Primary cilia indirectly activate ERK1/2 via ATP-dependent Ca^2+^ signals, while cilium-localized channels modulate mechanically induced Ca^2+^ entry and ERK activation [11,60,115,116,117].

Integrin-mediated mechanotransduction stimulates ERK1/2 via β1 integrin–Src–FAK signaling and downstream cascades such as PLCγ1/Rac1 or Pyk2, promoting cytoskeletal remodeling, proliferation, and matrix synthesis [46,118,119,120,121]. Aberrant ERK activation drives hypertrophy and OA progression, whereas pharmacologic or genetic ERK inhibition reduces MMP-13 expression. Mechanosensitive factors such as CITED2 can suppress ERK phosphorylation, decrease MMP-13, and activate autophagy, conferring chondroprotective effects in a context-dependent manner [122,123,124].

ERK also interacts with developmental pathways, including phosphorylation of GLI1/GLI2 to enhance Hedgehog signaling, and forms bidirectional networks with β-catenin to regulate proliferation, differentiation, and matrix homeostasis [125,126,127,128,129,130,131]. Collectively, MAPK–ERK, Wnt/β-catenin, and IHH constitute a coupled network driving hypertrophy, matrix degradation, and tissue degeneration [132]. Characterizing ERK–GLI and ERK–β-catenin axes across disease stages and cartilage zones is critical for understanding mechanical–developmental signal integration in OA.

### 4.4. TGF-β Pathway

TGF-β1 is sequestered in the PCM as a latent complex stabilized by LAP and LTBPs [133]. Mechanical stimuli, such as cyclic compression, release active TGF-β1, which binds TGFBR1/2 and activates SMAD2/3. Phosphorylated SMAD2/3 forms a heterotrimer with SMAD4, translocates to the nucleus, and regulates target gene transcription [134]. Integrin-mediated tension amplifies this activation, modulating chondrocyte metabolism and homeostasis.

Under physiological or low mechanical load, moderate TGF-β/SMAD2/3 activity maintains cartilage homeostasis by suppressing RUNX2 and MMP-13 and limiting nuclear β-catenin activity, restraining Wnt-driven hypertrophy [135,136,137]. Wnt/β-catenin reciprocally modulates SMAD2/3 nuclear translocation, allowing for dynamic fine-tuning of TGF-β signaling under mechanical and inflammatory conditions [137,138].

Excessive TGF-β/SMAD signaling in pathological mechanical or OA conditions triggers the SMAD2/3–HTRA1–DDR2–ERK/p38 MAPK cascade, upregulating MMP-13 and ADAMTS5 and accelerating ECM degradation and hypertrophy [139,140]. GLI proteins form complexes with SMAD2/3/4 and recruit p300/PCAF to enhance TGF-β target gene transcription, highlighting bidirectional GLI–SMAD crosstalk under OA-relevant mechanical stress [141,142]. Neutralization of TGF-β1 reduces MMP-13 expression and delays cartilage degeneration in OA models, emphasizing the pathological synergy between mechanical overload and aberrant TGF-β signaling [137].

### 4.5. Hippo–YAP Pathway

Hippo–YAP signaling serves as a central mechanotransductive hub governing cell proliferation, differentiation, and fate decisions. YAP and TAZ respond to ECM stiffness, PCM tension, and compressive loading by dynamic regulation of phosphorylation and subcellular localization, coupling mechanical inputs to context-specific transcriptional programs that preserve chondrocyte phenotypic homeostasis under physiological conditions [21,28,29,143,144].

In healthy cartilage, compliant PCM sustains LATS1/2 kinase activity, maintaining YAP/TAZ in a phosphorylated and predominantly cytoplasmic state, restraining hypertrophic differentiation programs [144]. YAP can physically associate with RUNX2 and suppress transcriptional activity, reducing hypertrophic marker expression such as type X collagen [145]. TAZ may cooperate with RUNX2 in specific contexts, indicating that the YAP/TAZ–RUNX2 axis is highly dependent on cell type, cytoskeletal tension, and microenvironmental cues [21,28].

In OA, inflammatory and stress-related signals modulate YAP/TAZ activity in a stimulus- and context-dependent manner, perturbing chondrocyte homeostasis and matrix turnover. IL-19 suppresses LATS1 expression and decreases YAP1 Ser127 phosphorylation, promoting YAP nuclear localization and disrupting cartilage regulatory programs [146]. Matrix stiffening enhances YAP/TAZ nuclear activity, contributing to phenotypic instability and loss of differentiated chondrocyte state [147,148]. Catabolic stimuli such as IL-1β increase YAP Ser127 phosphorylation while reducing nuclear translocation, highlighting dynamic regulation of Hippo signaling [149]. Aging-associated ER stress upregulates YAP expression and activates CTGF signaling, contributing to dedifferentiation and ECM degeneration [150].

Hippo–YAP signaling exhibits extensive crosstalk with IHH, Wnt/β-catenin, TGF-β/SMAD, and MAPK pathways [99,151,152,153,154,155,156,157]. Nuclear YAP enhances SMAD3-dependent transcription under increased ECM stiffness [148,153]. YAP/TAZ may interact with SMAD complexes to induce HTRA1 and activate DDR2–MMP-13, accelerating ECM degradation [154,155,156]. Overall, YAP/TAZ integrates mechanical, inflammatory, and degenerative inputs, representing a promising therapeutic target.

### 4.6. Notch Pathway

Notch signaling shows context-dependent roles. Moderate Notch activity maintains chondrocyte homeostasis and suppresses premature hypertrophic differentiation, whereas excessive activation under inflammatory or degenerative conditions upregulates type X collagen and MMP-13 via RUNX2-dependent mechanisms [158,159,160,161]. Direct evidence linking mechanical stimulus to Notch regulation is limited; however, the dual functional characteristics suggest sensitivity to mechanical microenvironment changes.

Notch and Hippo–YAP/TAZ signaling exhibit functional crosstalk. NICD can form complexes with YAP/TAZ and participate in transcriptional regulation via RBP-J or chromatin recruitment, while the YAP/TAZ–TEAD and Notch pathways influence downstream programs [24,162,163,164]. Shang et al. (2016) showed that NICD enhances BMP/SMAD signaling and upregulates p57, inducing cell cycle arrest and indirectly promoting hypertrophy [165]. In OA, Notch and YAP/TAZ are frequently elevated, potentially acting synergistically to drive hypertrophy and ECM degradation [24,166].

### 4.7. Other Pathways

#### 4.7.1. Interplay Between Mitochondrial Dynamics and Mechanical Signaling

Mitochondrial dynamics, the balance between fission and fusion, are central to energy metabolism, redox homeostasis, and apoptosis susceptibility [167,168]. Mechanical stress disrupts this balance, causing dysfunction and accelerating degeneration. Phosphorylation of Drp1 at Ser616 induces excessive fission and ROS production, activating NF-κB and MAPK, promoting MMP-13 expression, and driving hypertrophy [169,170,171,172,173]. Inhibition of Drp1 restores mitochondrial morphology, suppresses ROS, and delays hypertrophy [171,173]. Downregulation of Mfn2 impairs mitochondrial coupling, whereas restoration improves function and reduces oxidative/inflammatory signaling [174,175]. Aberrant mitophagy is also observed under excessive mechanical stimulation [176,177]. Activation of Nrf2–Parkin or Drp1 inhibition partially restores homeostasis and mitigates ECM degradation [173,178].

#### 4.7.2. Extracellular Vesicle-Mediated Intercellular Mechanotransduction

EVs mediate mechanically regulated intercellular communication in OA cartilage [179]. Mechanical stress alters EV abundance and cargo, influencing neighboring cells. EVs from stimulated chondrocytes carry miR-221-3p, affecting osteoblast CDKN1B and TIMP-3, impairing cartilage–bone crosstalk [180]. EVs enriched in YAP/TAZ or upstream regulators activate signaling in recipient cells, modulating differentiation [181,182,183]. Osteocyte-derived EVs with miR-23b-3p disrupt mitochondrial homeostasis and autophagy in chondrocytes [184]. EVs from inflammatory cells amplify pathological signaling, synergizing with mechanical stress to enhance hypertrophy and ECM breakdown [185,186].

#### 4.7.3. Adrenergic Receptor-Mediated cGMP–SLPI–RUNX2 Axis

α-Adrenergic receptor (α-AR) signaling regulates differentiation under mechanical stress. α2-AR activation suppresses chondrogenesis and promotes hypertrophy via cGMP-mediated SLPI upregulation, which activates RUNX2 and type X collagen [187,188,189,190]. SLPI knockout or inhibition enhances organoid resistance to degeneration [187,188,189,190]. RUNX2 drives chondrocyte transdifferentiation and ossification; knockout reduces hypertrophy and delays ossification [190,191]. α-AR antagonists block degeneration and promote hyaline-like regeneration [187].

#### 4.7.4. Dynamic Regulation of Epigenetic Modifications

Mechanical stimulation regulates chondrocyte differentiation via epigenetic mechanisms. HDAC4 shuttles to the nucleus under cyclic stretch/compression via PP2A-dependent dephosphorylation, suppressing RUNX2, IHH, MMP-13, and type X collagen, while upregulating SOX9, type II collagen, and PLK1 [189,192]. HDAC4 is downregulated in OA, and nuclear-enhanced HDAC4 mutants inhibit hypertrophy [193]. HDAC6 is upregulated in OA, modulating oxidative stress and autophagy; its inhibition with Tubastatin A reduces ROS, mitochondrial damage, apoptosis, and activates autophagy [194,195]. ncRNAs, including circRNA-MSR, induce TNF-α and inhibit ECM synthesis under stress, accelerating degeneration [196]. HDACs and ncRNAs crosstalk remains incompletely understood, representing a key direction for mechanotransduction–epigenetic research.

## 5. Anti-Hypertrophic Strategies via Physical Microenvironment Modulation

Chondrocyte hypertrophy represents a central pathological event in cartilage degeneration and osteoarthritis, and its modulation has emerged as a rational therapeutic target. While traditional intervention strategies have largely focused on biochemical or molecular pathways, a growing body of evidence demonstrates that, depending on measurement scale, tissue region, and OA stage, the physical microenvironment of cartilage, including matrix stiffness, viscoelasticity, architectural organization, and dynamic mechanical loading, can substantially influence chondrocyte phenotype and intracellular signaling. Accordingly, therapeutic concepts leveraging physical cues have gained increasing attention in both OA research and cartilage tissue engineering.

Importantly, anti-hypertrophic strategies targeting the physical microenvironment encompass two fundamentally distinct translational contexts. One context involves whole-joint, disease-modifying concepts aimed at attenuating aberrant mechanosensitive signaling in diffuse osteoarthritis, where pathological loading, altered tissue mechanics, and chronic inflammation collectively drive hypertrophic progression. The other context focuses on focal cartilage defect repair or regenerative applications rooted in tissue engineering frameworks, where engineered constructs are introduced to restore local cartilage structure and function. Although these two contexts share overlapping mechanobiological principles, their intended indications, evidence bases, and translational constraints differ substantially and therefore warrant explicit distinction.

**Figure 3:**
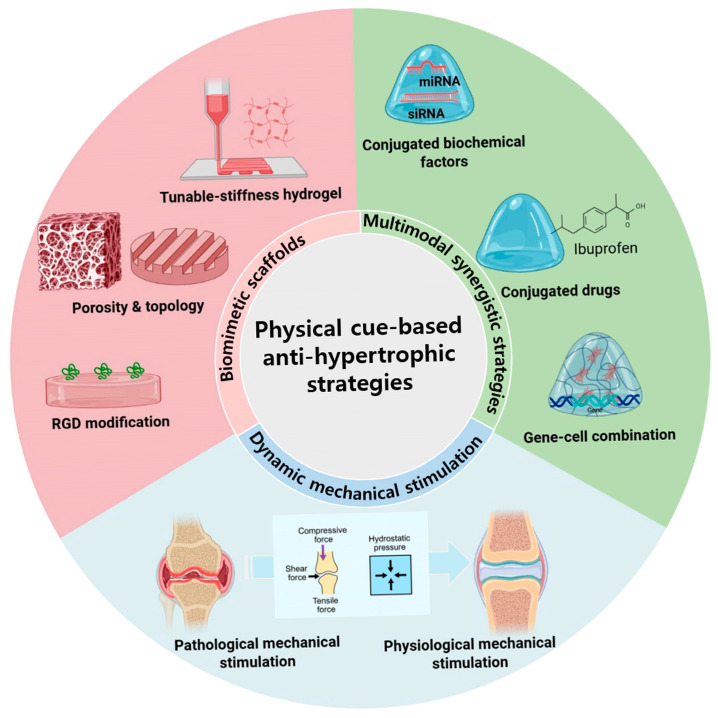
Overview of anti-hypertrophic strategies targeting the physical microenvironment. Anti-hypertrophic strategies aimed at regulating chondrocyte hypertrophy through physical microenvironmental cues can be broadly categorized into three conceptual approaches: (i) biomimetic scaffold-based microenvironmental optimization, (ii) dynamic mechanical stimulation, and (iii) multimodal synergistic regulation. Biomimetic scaffold strategies involve the design of matrices with tunable stiffness, controlled porosity, defined micro-/nanoscale topography, and surface chemical modifications to recapitulate key features of the native cartilage extracellular matrix. Dynamic mechanical stimulation encompasses compressive loading, shear stress, tensile strain, and hydrostatic pressure, which mimic physiological or pathological joint loading conditions and modulate mechanotransduction pathways. Multimodal strategies integrate physical cues with biochemical factors, drug delivery systems, and gene- and/or cell-based therapies to achieve coordinated regulation of hypertrophic and catabolic signaling. This schematic summarizes representative concepts derived primarily from in vitro and preclinical mechanobiological studies and is intended to provide a conceptual framework rather than a direct clinical roadmap. Abbreviations: RGD, arginine–glycine–aspartic acid; miRNA, microRNA; siRNA, small interfering RNA. Figure created with BioRender (licensed).

As illustrated in Figure 3, many anti-hypertrophic strategies have been developed and validated primarily under in vitro or preclinical conditions. Nevertheless, selected approaches may hold translational relevance. Biomimetic scaffolds designed to recapitulate key features of native cartilage ECM may be implanted to locally support chondrocyte homeostasis and suppress hypertrophic differentiation, particularly in focal defects. Controlled dynamic mechanical stimulation, such as targeted physiotherapy or joint loading regimens, may provide beneficial mechanical cues in vivo and contribute to the modulation of disease progression at the whole-joint level. Furthermore, integrating physical microenvironmental regulation with biochemical treatments, gene modulation, drug delivery, or cell-based approaches, including mesenchymal stem cells or extracellular vesicles, offers promising avenues for OA intervention. Together, these considerations illustrate how mechanistic insights derived from in vitro and preclinical mechanobiology can inform but should not be conflated with clinically actionable therapeutic strategies.

### 5.1. Physical Microenvironment Modulation in Regenerative Cartilage Repair

Within tissue engineering and regenerative medicine contexts, biomaterial-based modulation of the physical microenvironment has been extensively explored to suppress chondrocyte hypertrophy and promote stable cartilage formation. In vivo, articular chondrocytes reside in a mechanically compliant and hypoxic microenvironment that supports metabolic and phenotypic homeostasis and restrains terminal differentiation. In contrast, hypertrophic differentiation observed during in vitro expansion or repair is commonly associated with upregulation of type X collagen, RUNX2, and matrix mineralization, ultimately compromising the functional quality of regenerated cartilage.

Stiffness-tunable biomaterials have demonstrated anti-hypertrophic effects under defined experimental conditions, with outcomes that may depend on tissue region, material stiffness range, and OA stage. Softer PDMS substrates facilitate reversion of hypertrophic chondrocytes toward a chondrogenic phenotype, characterized by increased SOX9 and type II collagen expression and suppression of RUNX2 and type X collagen [197]. Similarly, GelMA–Fe2O3 hydrogels with adjustable stiffness preserve rounded chondrocyte morphology, maintain metabolic homeostasis, and support cartilage regeneration in vivo [198]. Beyond elastic stiffness, viscoelastic properties critically influence cell fate, as hydrogels with rapid stress relaxation attenuate ROCK-dependent apoptosis and promote ECM deposition and chondrogenic differentiation [199].

Microstructural features further modulate hypertrophic responses. Nanofibrous PLLA scaffolds combined with matrilin-3 suppress type X collagen and RUNX2 expression while preserving type II collagen synthesis [200], whereas triply periodic minimal surface architectures with defined geometries differentially regulate hypertrophic marker expression [201]. Surface chemical modification also contributes to phenotype regulation by modulating integrin-mediated signaling. Interaction between type II collagen and integrin β1 suppresses BMP–SMAD1 signaling and limits hypertrophic progression [202,203], while perturbation of integrin β1 signaling alters TGF-β–SMAD2/3-dependent chondrogenic programs [204].

Evidence Level and Translational Considerations: These regenerative strategies are primarily intended for focal cartilage defects or early degenerative lesions, rather than advanced, whole-joint OA. Evidence is strongest in in vitro and small-animal models, with select validation in large-animal studies; human clinical data remain limited. Translation is constrained by implant integration under complex joint loading, long-term phenotypic stability in inflammatory OA environments, and scalability for clinical-grade manufacturing.

### 5.2. Dynamic Mechanical Stimulation as a Modulator of Hypertrophy

Dynamic mechanical stimulation represents another effective approach to suppress hypertrophy and preserve a chondrogenic phenotype. Physiologically relevant mechanical cues, including cyclic compression, shear stress, tensile strain, and hydrostatic pressure, profoundly influence mechanotransduction pathways in chondrocytes and mesenchymal stem cells. Dynamic compression consistently reduces RUNX2 and type X collagen expression while promoting SOX9 and type II collagen synthesis in cell-laden hydrogels [205,206,207]. Hydrostatic pressure remodels chromatin architecture and suppresses pro-hypertrophic gene activity [208].

Combinatorial loading regimens further enhance chondrogenic outcomes. Shear stress applied together with dynamic compression promotes ECM deposition and chondrogenic differentiation [209], while cyclic equibiaxial tensile strain induces HDAC4 nuclear translocation, leading to repression of RUNX2 and type X collagen expression [189]. By tailoring loading magnitude, frequency, and mode, dynamic stimulation can partially restore physiological mechanosensing and attenuate hypertrophic signaling.

Evidence Level and Translational Considerations: Mechanically based interventions are most relevant to early-stage or mechanically driven OA, where cartilage architecture is partially preserved, and chondrocyte mechanosensitivity remains intact. Evidence derives from in vitro systems and small-animal models, with emerging clinical-adjacent rehabilitation studies. Limitations include heterogeneous joint loading, persistent inflammatory milieu, and difficulty achieving spatially controlled mechanical modulation in vivo. These strategies should be considered adjunctive or disease-modifying, not definitive OA therapies.

### 5.3. Integrated Multimodal Strategies and Translational Considerations

Chondrocyte hypertrophy in OA is initiated and sustained by aberrant mechanical cues, including altered matrix stiffness, abnormal shear stress, and uneven load distribution, which act as upstream triggers. These physical signals are sensed by integrins, primary cilia, and mechanosensitive ion channels and are subsequently integrated with biochemical and inflammatory cues to regulate hypertrophic gene expression. While isolated mechanical or biochemical inputs are often insufficient to drive hypertrophy independently, their coordinated interplay establishes a self-reinforcing pathological program.

Multimodal strategies that integrate physical microenvironment modulation with biochemical factors, drug delivery, or gene-based interventions have demonstrated anti-hypertrophic effects in vitro and in preclinical models, with translation to humans largely untested. Enzyme-responsive hydrogels releasing selective MMP-13 inhibitors [58] or dynamic collagen-based hydrogels delivering bioactive factors [210] suppress RUNX2 and type X collagen expression while promoting ECM accumulation. Mechanoresponsive carrier systems enable controlled release of therapeutic agents in response to mechanical loading, maintaining cartilage homeostasis without continuous external stimulation [211,212,213,214]. Anti-inflammatory hydrogels delivering cartilage organoids provide both mechanical support and immunomodulatory effects, facilitating regeneration in inflammatory environments [215].

Mechanical preconditioning of mesenchymal stem cells under dynamic compression enhances in vivo cartilage repair and preserves chondrogenic potential through activation of intracellular transcriptional programs [216]. Scaffold-mediated gene delivery further enables spatiotemporal control of growth factor or anti-inflammatory gene expression, synergizing with mechanical cues to support precision regenerative therapy [217].

Evidence Level and Translational Considerations: These integrated multimodal strategies are primarily preclinical. Translation to humans is constrained by system complexity, regulatory barriers, and the challenge of synchronizing mechanical, biochemical, and genetic inputs in vivo. They represent a mechanistic framework guiding therapeutic development rather than a direct clinical roadmap.

## 6. Future Perspectives in OA Therapy

Research in cartilage mechanobiology and OA therapy is increasingly shifting from isolated biological mechanisms toward multidimensional, integrative strategies that combine advanced biomaterials, cellular mechanobiology, and emerging computational technologies. This transition reflects growing recognition that chondrocyte hypertrophy and cartilage degeneration are governed by tightly coupled mechanical, biochemical, and inflammatory cues operating in a context- and stage-dependent manner [218,219,220,221,222].

Extracellular vesicles have emerged as important mediators of intercellular communication within mechanically active cartilage microenvironments. Under defined in vitro and preclinical conditions, EVs carrying signaling molecules such as Wnt5a and Wnt5b have been shown to activate YAP signaling in recipient cells and modulate hypertrophic differentiation [185,223]. However, whether chondrocyte-derived EVs directly transport YAP or TAZ proteins, and whether such transfer results in sustained activation of hypertrophic programs across distinct cartilage zones and OA stages, remains unresolved. Systematic characterization of EV cargo, mechanosensitivity, and spatiotemporal signaling dynamics will be essential to establish their mechanistic and translational relevance.

From a biomaterials perspective, smart hydrogels with tunable viscoelasticity and dynamically adjustable mechanical properties offer promising platforms to mimic the native cartilage microenvironment and modulate mechanosensitive signaling [224,225,226]. Hyaluronic acid-based dynamic hydrogels and programmable three-dimensional bioprinting technologies may enable spatially organized regeneration of cartilage and osteochondral interfaces [218]. Nevertheless, most supporting evidence remains preclinical, and translation to human OA requires careful consideration of joint-level loading heterogeneity, disease stage, and tissue-specific mechanical properties.

Artificial intelligence and machine learning approaches are increasingly applied to OA phenotyping, prognosis prediction, and patient stratification [227,228,229], while wearable biosensors and virtual reality-assisted rehabilitation systems enable real-time monitoring of joint function and mechanical loading [230,231,232,233]. At present, these technologies should be viewed primarily as enabling tools rather than direct anti-hypertrophic interventions. Their successful integration into clinical practice will depend on validation under physiologically relevant loading conditions, across diverse patient populations, and with long-term outcome assessment.

Looking forward, effective OA therapies will likely require integration of mechanobiological insights, advanced biomaterials, and computational approaches within a precision-medicine framework. Linking mechanical forces, mechanotransduction pathways, gene regulation, and functional tissue regeneration in a spatiotemporally controlled manner remains a major challenge. Addressing this challenge will be critical for translating mechanobiological discoveries into individualized, clinically actionable strategies for cartilage repair and OA therapy.

## 7. Conclusions

Physical cues act as central determinants of chondrocyte hypertrophy, regulating mechanosensing, transcriptional programs, and ECM remodeling via multiple mechanotransduction pathways. Advances in understanding ECM mechanical properties, mechanosensitive receptors, and core signaling modules, including canonical Wnt/β-catenin signaling that predominantly promotes hypertrophic differentiation and cartilage catabolism, as well as non-canonical Wnt pathways that regulate cytoskeletal organization and context-dependent responses, together with YAP/TAZ and mechanosensitive ion channels, have revealed molecular nodes with potential therapeutic relevance.

Emerging biomaterials with tunable viscoelasticity, dynamic responsiveness, and precise biochemical presentation, combined with multimodal mechanical stimulation strategies, offer promising avenues under defined experimental conditions to restore physiological mechanosensing and suppress chondrocyte hypertrophy. Integration of these approaches with targeted molecular modulation and engineered microenvironments may enhance the efficacy and durability of anti-hypertrophic interventions. Given that joint loading patterns vary substantially across age groups, body mass index categories, and OA stages, mechanobiology-informed therapies are unlikely to be universally applicable across all patients. Accordingly, future therapeutic strategies should be developed within a precision medicine framework, accounting for patient-specific mechanical environments, cartilage material properties, and mechanosensitive signaling states. Elucidating the spatiotemporal dynamics of mechanotransduction and developing tools capable of precise control of mechanical inputs will be essential for translating mechanobiological insights into effective, individualized clinical strategies for cartilage repair and OA therapy.

## Figures and Tables

**Table 1:** Integrative overview of ECM biophysical cues, mechanosensors, and chondrocyte hypertrophy in OA.

Physical Cue (Zone/Stage/Scale Context)	Mechanosensor/Interface	Key Downstream Node(s)	Major Hypertrophy/Catabolic Outputs	Evidence Strength/Translational Note	References
ECM stiffness (PCM vs. bulk; superficial early OA vs. deep late OA)	Integrins/FAK/cytoskeleton–nucleus	RhoA/ROCK, YAP/TAZ	Type X collagen, MMP-13	In vitro, small animal; human cartilage explants—context-dependent, caution: local micromechanics vs. bulk tissue	[15,21,48,49,50,51,52,53,54]
ECM viscoelasticity (zone-dependent; altered in OA)	Integrins, Piezo1/TRPV4, primary cilia	NF-κB, Ca^2+^ signaling, YAP/TAZ	MMP-13, ADAMTS5, hypertrophy	In vitro hydrogels, human cells—translational limitation: tissue depth, loading conditions	[32,55,56,57,58,59,60,61]
Shear/interstitial fluid flow (localized pericellular flow; superficial zones)	Primary cilia, integrins	Ca^2+^ influx, MAPK, NF-κB	Type X collagen, MMP-13	In vitro, ex vivo cartilage explants—mainly mechanistic	[60,61]
ECM porosity/pore size (nano-to-micro, gradient by zone)	Primary cilia, integrins	HIF-1α/HIF-2α, RUNX2	Hypertrophy, MMP-13	In vitro, scaffold constructs—tunable pore size; varies by tissue engineering application	[48,50,58,59,62,63,64,65,66,67,68,69,70,71,72,73,74]
ECM topography (superficial vs. middle vs. deep zones)	Integrins/focal adhesions	RhoA/ROCK, Rac1/Cdc42	Type X collagen, MMP-13	In vitro—mechanistic; translation to 3D tissue requires scaffold mimicry	[74,75,76,77,78]
Surface roughness (nano/micro; OA fissures vs. engineered surfaces)	Integrins/FAK/ERK/ILK	MAPK, β-catenin	Type X collagen, MMP-13	In vitro—biomaterials studies; in vivo relevance under investigation	[79,80,81]
Charge distribution/GAG content (fixed negative charge loss in OA; superficial > deep)	Piezo/TRPV4, integrins	Ca^2+^ signaling, calpains	ECM degradation, MMP-13	In vitro—ionic microenvironment affects mechanosensitive channels; in vivo relevance under investigation	[33,40,82,83,84,85,86,87]
Osmotic/swelling cues (zone-dependent; hypoxic gradients)	Primary cilia, integrins	HIF-1α/HIF-2α, NF-κB	Hypertrophic markers, MMP-13	In vitro, tissue explants—translation limited by oxygen/nutrient gradients in whole-joint	[60,61,66,67,68,69]

## Data Availability

No new data were created or analyzed in this study. Data sharing is not applicable to this article.

## Abbreviations

The following abbreviations are used in this manuscript:

α-AR	α-adrenergic receptor
ADAMTS5	A disintegrin and metalloproteinase with thrombospondin motifs 5
ALP	Alkaline phosphatase
CaMKs	Ca^2+^/calmodulin-dependent protein kinases
CDKN1B	Cyclin-dependent kinase inhibitor 1B
C/EBPβ	CCAAT/enhancer-binding protein-β
cGMP	Cyclic guanosine monophosphate
circRNA-MSR	Mechanical stress-related circRNA
CTGF	Connective tissue growth factor
CITED2	CBP/p300-interacting transactivator with ED-rich tail 2
Drp1	Dynamin-related protein 1
ECM	Extracellular matrix
EVs	Extracellular vesicles
FAK	Focal adhesion kinase
FGF2	Fibroblast growth factor 2
GAGs	Glycosaminoglycans
GTPase	Guanosine triphosphatase
HDACs	Histone deacetylases
HTRA1	High-temperature requirement A serine peptidase 1
IFT	Intraflagellar transport
IHH	Indian hedgehog
IL-1β	Interleukin-1β
ILK	Integrin-linked kinase
LAP	Latency-associated peptide
LTBP	Latent TGF-β binding protein
MAPK	Mitogen-activated protein kinase
ERK	Extracellular signal-regulated kinase
Mfn2	Mitofusin 2
miRNAs	MicroRNAs
MMPs	Matrix metalloproteinases
MSCs	Mesenchymal stem cells
NF-κB	Nuclear factor-κB
NICD	Notch intracellular domain
OA	Osteoarthritis
PCM	Pericellular matrix
PLCγ1	Phospholipase C γ1
PLK1	Polo-like kinase 1
PP2A	Protein phosphatase type 2A
RhoA/ROCK	RhoA/Rho-associated coiled-coil kinase
ROS	Reactive oxygen species
RUNX2	Runt-related transcription factor 2
SLPI	Secretory leukocyte protease inhibitor
SOX9	Sex determining region Y box protein 9
TGF-β	Transforming growth factor-β
TGFBR1/2	Transforming growth factor-β receptors I/II
TIMP-3	Tissue inhibitor of metalloproteinases 3
TM	Territorial matrix
TNF-α	Tumor necrosis factor-α
TRPV4	Transient receptor potential vanilloid subtype 4
YAP/TAZ	Yes-associated protein/transcriptional coactivator with PDZ-binding motif
